# Effects of furosemide on the hearing loss induced by impulse noise

**DOI:** 10.1186/1745-6673-6-14

**Published:** 2011-05-08

**Authors:** Cahtia Adelman, Jeffrey M Weinberger, Leonid Kriksunov, Haim Sohmer

**Affiliations:** 1 Speech & Hearing Center, Hadassah University Medical Center, POB 12000, Jerusalem 91120, Israel; 2 Dept. of Otolaryngology and Head & Neck Surgery, Hadassah University Medical Center, POB 12000, Jerusalem 91120, Israel; 3 Dept. of Communication Disorders-Hadassah Academic College, POB 1114, Jerusalem 91010, Israel; 4 Dept. of Physiology; Institute for Medical Research - Israel-Canada; Hebrew University-Hadassah Medical School, POB 12272, Jerusalem 91120, Israel

**Keywords:** impulse noise, noise induced hearing loss, protection, cochlear amplifier, outer hair cell motility, active mechanical displacements, free radicals, furosemide

## Abstract

**Background:**

The permanent hearing loss following exposure to intense noise can be due either to mechanical structural damage (tearing) caused directly by the noise or to metabolic (biochemical) damage resulting from the elevated levels of free radicals released during transduction of the sound overstimulation. Drugs which depress active cochlear mechanics (e.g. furosemide and salicylic acid) or anti-oxidants (which counteract the free radicals) are effective in reducing the threshold shift (TS) following broadband continuous noise. This study was designed to determine whether furosemide can reduce the TS following exposure to impulse noise, similar to its action with continuous broadband noise.

**Methods:**

Shortly after furosemide injection, mice were exposed to *simulated *M16 rifle impulse noise produced by different loudspeakers and amplifiers in different exposure settings and, in other experiments, also to *actual *M16 rifle shots.

**Results:**

Depending on the paradigm, the *simulated *noises either did not produce a TS, or the TS was reduced by furosemide. The drug was not effective in reducing TS resulting from *actual *impulse noise.

**Conclusion:**

*Simulated *M16 rifle impulse noise may not truly replicate the rapid rise time and very high intensity of *actual *rifle shots so that the TS following exposure to such noise can be reduced by these drugs. On the other hand, *actual *M16 impulse noise probably causes direct (frank) mechanical damage, which is not reduced by these drugs.

## Background

Noise induced hearing loss (NIHL) affects many people in the world. The source of the noise can be industrial, recreational or military [1]. Therefore attempts have been made to prevent and alleviate the resulting impairment. These attempts include educational efforts [2], use of mechanical ear protecting devices [3] and pharmaceutical agents [4,5]. The types of noise can be broadly divided into two categories: continuous noise such as from personal music players, and impulse noise, for example that resulting from firearms. Much research has focused on the possible administration of drugs which could prevent the damage resulting from the exposure to continuous noise. Such research, besides suggesting drugs which could alleviate the NIHL, also provides insight into the possible mechanism of the NIHL caused by exposure to continuous noise. For example, it has been shown that if one administers, just before (but not after) a continuous noise exposure, drugs which reversibly reduce the magnitude of the active mechanical displacements produced in the cochlea in response to sound (temporarily depressing the cochlear amplifier, with reduced outer hair cell motility and decreased active basilar membrane displacement), the resulting permanent threshold shift (PTS) is significantly smaller than that in animals given saline (mean PTS was 15 dB smaller with salicylic acid [6]; mean PTS was 12 dB smaller with furosemide [7]). This result, together with the finding that several anti-oxidant drugs administered before and after a continuous noise exposure (for example, salicylate and trolox [4,5,8]) are also effective in protecting the ear, provides evidence that the hearing loss following exposure to continuous noise is due to the generation of elevated levels of reactive oxygen and nitrogen species (ROS and RNS), free radicals, which then cause metabolically (biochemical) originating damage to vital structures in the cochlea [9]. The production of elevated levels of ROS is due to the higher metabolic demand needed to maintain the cochlear electrical-chemical gradients required for transduction during the noise exposure. The elevated ROS levels are a direct result of the higher metabolic demand. Therefore, in the presence of drugs which reversibly depress the active mechanical displacements, there is reduced metabolic demand, producing lower levels of ROS, and in this case of reduced levels of ROS, the administration (in addition to the furosemide) of anti-oxidant drugs which counteract the ROS does not provide further protection [10].

On the other hand, the mechanism of NIHL following exposure to impulse noise is likely more complicated since impulse noise is intermittent, it reaches very high sound pressures with a very rapid rise time [11] and is of short duration. Therefore the NIHL seen following impulse noise can be the product of direct (frank) mechanical disruption of tissues (tearing) and/or indirect metabolically originating tissue damage resulting from production of elevated levels of ROS [12], as seems to be the case with continuous noise. In this study, we attempted to gain insight into the possible mechanism(s) of the NIHL following exposure to impulse noise by administering one of the drugs (furosemide) which reversibly depresses the cochlear amplifier and has been found [7] to provide protection from continuous broadband noise. The drug was administered in a single injection just before the exposure to the impulse noise, similar to the timing of injections found to provide protection from continuous broadband noise. The type of impulse noise chosen for study was the M16 rifle in use by the armed forces of many countries. The first stage of the study involved attempts to adapt the M16 impulse noise exposure to laboratory conditions by making use of a *simulated *M16 impulse electrical waveform obtained from a sound effects site on the Internet. This electrical waveform was applied to various types of amplifiers, loud speakers and exposure conditions in order to deliver the simulated M16 rifle shots to mice under laboratory conditions. The results of this phase of the experiment led us to the second stage in which mice were exposed to *actual *M16 rifle shots during target practice sessions in an open firing range.

## Methods

### General outline of experimental paradigm

The experiments were conducted on seven-week-old albino male mice (body weight 35-45 grams) of the normal Sabra strain obtained from Harlan, Israel. Auditory thresholds before and after the exposure to the impulse noise were assessed by recording the auditory nerve brainstem evoked response (ABR) thresholds to broadband clicks (which deliver a wide range of frequencies) and 8000 Hz tone bursts (TB) under anesthesia. These stimuli were chosen in order to enable uniformity and comparison with the experiments conducted in this laboratory with continuous noise exposure and furosemide [7,10]. The ABR thresholds in these mice were similar to those in fat sand rats (Psammomys obesus) and to the behavioral thresholds of normal hearing humans to the same broadband clicks delivered by the same insert earphones. Only animals with ABR thresholds to broadband clicks of 65 dB peak equivalent (pe) SPL or better were used in the study. There was no change in threshold over the time duration (one week) of this experiment (i.e. control for aging) [6]. The next day, when anesthesia had worn off, a single injection of 100 mg/kg of furosemide was administered intraperiteoneally (IP) to the mice of the experimental group and the mice of the control group were injected (IP) at the same time with a similar volume of saline. All mice, experimental (furosemide) and control (saline) together, were exposed to M16 impulse noise (either simulated in the laboratory, or actual) one hour after the injections, at the time of maximal cochlear amplifier depression, as indicated by elevated hearing thresholds following furosemide injection in earlier experiments [7]. ABR thresholds to click and to 8000 Hz tone bursts were recorded again after a week and compared to the initial thresholds, to determine resulting threshold shifts (TS). Initial thresholds in both the control and the experimental groups were compared. As no significant difference was found between these initial thresholds (two-tailed t-tests), the statistical evaluation (again using two-tailed t-tests) was then conducted on the thresholds determined a week after the noise exposure

### Anesthesia

The ABR thresholds to clicks and to 8000 Hz tone bursts were determined in mice anesthetized with Avertine 11.25 mg/kg injected intraperitoneally (IP). Additional anesthesia was administered if necessary to maintain areflexia.

### Auditory Brainstem Response (ABR)

Stimuli were presented to the left ear through an insert earphone, and ABR was recorded between subdermal needle electrodes at vertex and chin, with a ground electrode in the left hindlimb, using a Biologic Navigator Pro evoked potential system (Bio-logic Systems Corp., Mundelein, Il., USA). Thresholds were recorded to two types of stimuli: alternating polarity broadband clicks and alternating polarity 8000 Hz tone bursts (Blackman ramp, with a rise/fall time of 0.5 msec and a plateau of 5 msec). The stimuli were presented from a maximal intensity of 120 dB pe SPL and decreased to below threshold in 5 dB steps. The responses were filtered (band pass 300-3000 Hz), amplified, and 128-256 responses were averaged. The lowest stimulus intensity at which repeatable components of ABR (usually the first wave - the compound action potential of the auditory nerve) were obtained in at least two recordings was defined as threshold.

All of the experiments were evaluated and approved by the Hebrew University - Hadassah Medical School Animal Care and Use Committee.

### *Simulated *M16 rifle impulse noise

The electrical waveform of the M16 firing was obtained from a sound effects Internet site [13]. Preliminary experiments were conducted in order to assess the optimal combination of amplifiers, loud speakers, number of firings (each click of the mouse of the computer provided a single firing), their sound intensity and the mode of their delivery to the experimental animals which would provide a TS when recording a week later. The desired TS was one which could be measured, yet was not too great for possible reduction (protection) by the drug administered (to avoid a "ceiling effect").

#### Initial experimental presentations of simulated M16 noise which did not produce a TS a week later

##### Experiment I-Methods and Results

These experiments included presenting the simulated M16 noise by standard computer speakers placed on top of the cage of the animals. The peak intensity of each M16 firing which was measured with a Bruel & Kjaer precision integrating sound level meter (type 2218) was 123 dB SPL, but did not produce a TS when recording a week later. An additional preliminary experiment involved a power amplifier (50W Yamaha EM120 power amplifier) with a very large (89.5 cm high; 47 cm wide; 43.5 cm depth) loud speaker (Electro-Voice ECS 15-3 300 W speaker-40 cm diameter of diaphragm), providing a peak sound level of about 155 dB SPL. Nevertheless, 700 firings of such a simulated M16 rifle did not produce a TS in recordings a week later in two fat sand rats (Psammomy obesus) and this noise exposure paradigm was not applied further.

#### Experiments with simulated M16 exposure which produced a TS a week later

##### Experiment II-Methods

More powerful computer speakers (a pair of 20W computer speakers; Yamaha powered speaker model DM-01) produced 135 dB SPL peak noise. Eleven mice were injected with furosemide and a control group of 8 mice were injected with a similar volume of saline and then all 19 mice, awake, were exposed together, an hour later, to the 135 dB SPL simulated M16 impulse noise. The speakers were placed on the top of the cage, and 120 firings were presented, dispersed randomly over a period of six minutes.

##### Experiment II-Results

Because t-tests showed that there was no significant difference between the initial thresholds in the saline group (mean thresholds: to broadband clicks 57.5 ± 7.1 dB pe SPL, to 8000 Hz TB 58.1 ± 10.0 dB pe SPL) and the group which would be receiving furosemide (mean thresholds: to click 58.2 ± 6.4 dB pe SPL, to 8000 Hz TB 62.7 ± 12.1 dB pe SPL) before the exposure to noise, the statistical evaluations and comparisons were conducted on the thresholds determined a week after the simulated M16 noise exposure. The thresholds to clicks and to 8000 Hz tone bursts in the saline control group were significantly elevated a week later by the simulated noise exposure (to click 69.4 ± 15.5 dB pe SPL; P < 0.05, to 8000 Hz TB 73.1 ± 13.4 dB pe SPL; P < 0.005; one-tailed t-tests), i.e. the simulated noise caused a significant threshold shift in the control group (a mean TS of 11.9 dB [± 18.3] in response to clicks and 15.0 dB [± 11.7] with 8000 Hz tone bursts). On the other hand, in the furosemide group, the threshold (to click 56.4 ± 6.0 dB pe SPL, to 8000 Hz TB 58.6 ± 9.5 dB pe SPL) was not significantly different from the initial threshold (i.e. there was no TS in this furosemide group, meaning total protection). Thus in this experiment with the more powerful computer speakers which produced a TS of 12-15 dB in mice, the furosemide provided protection from the simulated M16 impulse noise.

##### Experiment III-Methods

Exposure to simulated M16 impulse noise in a specially constructed exposure chamber. In order to enhance the acoustic energy reaching the mice, and to prevent dispersion of the impulse noise to a large sound field, a small volume exposure box was designed (dimensions 31 cm length; 20 cm width; 32 cm depth). The simulated M16 was amplified by a NAD C300 stereo integrated amplifier. The speaker (Focal JM Lab Chorus 705V) fit snugly in the upper part of the box and the animal enclosure in the lower section. The diaphragm of the speaker was 16 cm above the floor of the mice enclosure, so that the net volume of the exposure chamber was 9920 cm^3^. Mice in one group (n = 10) received a single of injection of furosemide one hour before exposure to 10 computer simulated M16 shots, estimated (with sound level meter) at 155 dB peak SPL. Mice of the other group (control, n = 10) were injected with a similar volume of saline at the same time, and exposed to the same noise together with the furosemide group.

##### Experiment III - Results

Mean initial broadband click ABR threshold was 59.0 ± 6.15 dB pe SPL in the saline group and 60.5 ± 5.5 dB pe SPL in the group which would be receiving furosemide (two-tailed t-test, no significant difference, p = 0.57). Mean initial ABR threshold to 8000 Hz tone burst was 64.5 ± 14.6 dB pe SPL in the saline group and 62.0 ± 14.8 dB pe SPL in the furosemide group (two-tailed t-test, no significant difference, p = 0.71).

Mean click ABR threshold one week after exposure to the simulated M16 noise was 81.5 ± 18.4 dB pe SPL in the saline group and 62.5 ± 7.9 dB pe SPL in the furosemide group. Comparison of click thresholds before and after exposure to noise in the saline group showed a significant threshold shift (2-tailed, paired t-test, p < 0.005). Thresholds before and after exposure to noise were not significantly different in the group injected with furosemide (2-tailed, paired t-test, p = 0.22); i.e. total protection.

Mean 8000 Hz TB ABR threshold one week after exposure to noise was 81.0 ± 18.7 dB pe SPL in the saline group and 63.0 ± 14.6 dB pe SPL in the furosemide group. Comparison of TB thresholds before and after exposure to noise in the saline group showed a significant threshold shift (2-tailed, paired t-test, p < 0.05); i.e. noise exposure induced a significant threshold shift. There was no significant difference between thresholds before and one week after exposure to noise in the furosemide group (2-tailed, paired t-test, p = 0.8); i.e. total protection.

Comparison of the two groups (saline vs furosemide) revealed a significant difference between thresholds to click (two-tailed t-test, p < 0.05) and to tone bursts (two-tailed t-test, p < 0.05) one week after exposure to noise. Thus in this preliminary experiment with simulated M16 impulse noise in a specially constructed exposure chamber, furosemide was effective in protecting the mice. Based on these findings, the next stage of the study involved complementing the simulated M16 experiments with exposure of mice to actual M16 rifle shots during a target practice session in an outdoor rifle firing range.

#### *Actual *M16 rifle impulse noise (outdoor firing range)

##### Experiment IV - Methods

Mice in one group (n = 8) received a single injection of furosemide one hour before exposure to 10 M16 shots at a distance of 5 meters, estimated with sound level meter at approximately 155 dB peak SPL. Mice of the other group (control, n = 8) were injected with a similar volume of saline at the same time, and exposed to the same noise together with the furosemide group.

##### Experiment IV - Results

Mean initial click ABR threshold was 56.9 ± 7.0 dB pe SPL in the saline group and 56.3 ± 6.4 dB pe SPL in the group which would be receiving furosemide (two-tailed t-test, no significant difference, p = 0.86). Mean initial ABR threshold to 8000 Hz tone burst was 59.38 ± 7.76 dB pe SPL in the saline group and 55.6 ± 4.2 dB pe SPL in the furosemide group (two-tailed t-test, no significant difference, p = 0.25).

Mean broadband click ABR threshold one week after exposure to noise was 70.6 ± 12.7 dB pe SPL in the saline group and 72.5 ± 6.6 dB pe SPL in the furosemide group. Comparison of click thresholds before and after exposure to noise in the saline group showed a significant threshold shift (1-tailed, paired t-test, p < 0.05). A significant (1-tailed, paired t-test, p < 0.005) threshold shift was also found in the group injected with furosemide.

Mean 8000 Hz TB ABR threshold one week after exposure to noise was 73.1 ± 13.9 dB pe SPL in the saline group and 80.6 ± 14.5 dB pe SPL in the furosemide group. Comparison of TB thresholds before and after exposure to noise in the saline group also showed a significant threshold shift (1-tailed, paired t-test, p < 0.05), and a significant (1-tailed, paired t-test, p < 0.005) threshold shift was found in the furosemide group as well. Thus exposure to actual M16 firing produced a threshold shift both in the furosemide and in the saline control groups.

Comparison of the two groups (furosemide to saline) showed that there was no significant difference between thresholds to click or to tone bursts one week after exposure to noise (two-tailed t-tests). Mean threshold shift to broadband clicks was 13.8 ± 15.5 dB in the saline group and 16.3 ± 9.9 dB in the furosemide group, with no significant difference between them (two-tailed, p = 0.7). Mean threshold shift to TB was 13.8 ± 19.6 dB in the saline group and 25.0 ± 15.1 dB in the furosemide group, again with no significant difference between them (two-tailed, p = 0.2).

The results of these experiments (group, intensity and rise time of the impulse noise, the degree of the threshold shift and possible protection by furosemide) have been summarized in Table 1.

**Table 1:** Summary of experimental groups, intensity and rise time of impulse noise, degree of threshold shift and possible protection by furosemide

	Noise				
				
	Intensity (dB SPL)	Presumed rise time (μsec)	TS (dB)	Furosemide protection	Explanation (see text for details)
**Simulated MI6; sound effects site**					

**Experiment** **Ia - Computer speaker **(mice)	123	>88	NS	NA	Intensity too low

**Ib - Power amplifier, very large speaker **(Psammomys)	155	>>88	NS	NA	Rise time too slow

**II - 20W speaker **(mice)	135	>88	11.9	Total	Active displacements reduced by furosemide

**III - Small exposure chamber **(mice)	155	>88	22.5	Total	Active displacements reduced by furosemide

**Actual M16; firing range**					

**IV - Actual M16; firing range **(mice)	155	88	13.7	None	Tearing damage; furosemide not effective

NS - not significant

NA - not applicable

## Discussion

Because furosemide, in a single injection just before exposure to continuous *broadband *noise, leads to a smaller NIHL i.e. protection, this study was designed to determine whether such would be the case with *impulse *noise. Impulse noise is defined as "a short duration sound that is characterized by a shock wave having nearly instantaneous rise time" [14]. In the present report, the impulse noise chosen for study was that of the firing of an M16 rifle. Because exposing animals to actual M16 firings is not practical in the laboratory, we began with *simulated *M16, obtaining the electrical waveform from a sound effects site and transducing it with several different types of amplifiers, speakers and exposure settings. Others [15-19] have also used computer simulations for impulse noise, for the same practical reasons. In the present study, the ability of furosemide to protect from exposure to *actual *M16 firing was also assessed. It is difficult to measure impulse noise (which is characterized by extremely high levels, rapid rise times and short durations) accurately using conventional sound level meters (which are designed to function only up to about 140 dB SPL), and there are as yet no standards or norms for measurement [11,20,21]. Therefore, in the present study, the peak levels of the impulse noise had to be estimated. Using specially designed equipment, it has been shown [11] that the impulse noise from actual firing of an M16 rifle reaches a peak level of 165 dB SPL with a rise time of 88 μsec. In the present study, initial experiments showed that simulated M16 is not always effective in producing a threshold shift. In further experiments with different exposure procedures (amplifiers and speakers), the simulated M16 did produce a threshold shift in measurements a week after the exposure to noise, and furosemide was effective in reducing this threshold shift, similar to the protection it provides with continuous broadband noise. However, when the experiment was repeated using *actual *M16 shots in a firing range, the furosemide failed to provide protection. This seems to be similar to the ineffectiveness of the anti-oxidant NAC in reducing the permanent threshold shift and hair cell loss in the ear of chinchillas exposed to high kurtosis noise (i.e. a combination of high intensity impulse noise and continuous noise) presented for a long duration (eight hours per day for five days) [22], whereas it has been reported that NAC can reduce the permanent threshold shift due to continuous noise [18].

Thus there are discrepancies between the magnitudes of the threshold shift in these different experiments, even though the threshold shift was assessed in a uniform manner, i.e. in the same species, same drug, with the same ABR evaluation. These apparent discrepancies between simulated impulse noise which did not cause a threshold shift, simulated impulse noise producing a threshold shift which was reduced by furosemide and actual impulse noise causing a threshold shift which was not reduced by furosemide, require further thought. Actual impulse noise is intermittent (and not continuous as is broadband noise); and even during these transient bursts of noise, the intensity is high at the onset of each such burst and then rapidly declines. Each explosive firing reaches a very high peak level with a very rapid rise time. The rapid rise to high peak levels can cause direct (frank) mechanical damage to inner ear structures [1] and it is not likely that furosemide, which provides protection when administered just before the noise by reducing active OHC displacements, could provide protection from this component of the noise. In addition, the high peak levels can also induce biochemical (metabolic) damage resulting from the elevated release of ROS accompanying the greater metabolic demands of transduction during the high level noise exposure. This component of the impulse noise threshold shift can be effectively reduced by furosemide, as during exposure to continuous broadband noise.

In those initial simulated impulse noise experiments in which a threshold shift (assessed a week after exposure to noise) was not induced, it is likely that the intensity was not sufficiently high (smaller speakers), and/or that the rise time was slower than that in actual M16 rifle firings (since the simulation was lacking the explosive firing component), and the total duration of the impulse noise resulting from shots dispersed over some time period was less than that from continuous noise presented over the same time period. Thus it is likely that the simulated impulse noise presented intermittently (not continuously) did not produce excessive ROS levels, and the intrinsic levels of anti-oxidants were able to counteract them.

On the other hand, furosemide did provide protection in one of the simulated M16 experiments, where peak intensity was 155 dB SPL. In this case, there is reason to believe that the peak intensity and the rise time of the small speaker used to produce the simulated M16 impulse did not accurately replicate those of an actual M16 firing (88 μsec) due to the absence of an initial supersonic shock wave, accompanying the explosive force of the firing, and that the small dimensions of the exposure box caused reverberations (reflections) from the surfaces of the box with various phases and with a short latency which would degrade, smear and reduce the rise time of the impulse reaching the ear [20,21]. In such a situation, the simulated impulse noise would be dominated by the high intensity noise component (which can be reduced by the furosemide) and not by the rapid rise time component (which was degraded). Similarly, in a simulated noise experiment in which fat sand rats were exposed to 700 shots from a very large speaker (which did not produce a threshold shift), the very large diaphragm of the speaker was not able to "follow" the rapid rise time of the electrical waveform so that this too did not conform to the definition of impulse noise, which includes rapid rise time.

Thus, when the intensity and the rapid rise time of the impulse noise are compromised (degraded) by the experimental conditions (e.g. a speaker that is unable to replicate the intensity and rise time of an actual M16 firing, or reverberations causing degradation of the rapid rise time), either there is no threshold shift or the threshold shift, induced by the high intensity noise component, can be reduced by furosemide. Other research groups have also delivered simulated impulse noise by means of a closed sound system through a narrow tube [17,23] i.e. a small, cone shaped horn placed in the ear canal of animals, or very close to the ear [15,24] and it has been reported that different drugs and agents (the antioxidant NAC, Mg, conditioning) provided protection from the impulse noise. The closed, narrow bore impulse noise delivery system was used in order to conserve the impulse noise sound energy without dispersion to a large volume. However it is likely that the small horn also led to reverberations (reflections), with degradation of the rapid rise time, so that the antioxidants could reduce NIHL from impulse noise by neutralizing the resulting ROS. The possibility that some protection against impulse noise which has all of the attributes of actual impulse noise (reaching a very high intensity, within a very short rise time) can be provided by these drugs leads to the suggestion that such a noise also induces ROS, and not only direct mechanical (frank) damage (tearing), as has been suggested [1,12].

It has been shown that NAC can protect against *simulated *impulse noise [17,18]. Therefore, consideration should be given to further research with *actual *M16 impulse noise, in which the protection provided by antioxidants would be determined, as in the present study with furosemide, using a similar experimental paradigm. The protective effect of antioxidants and furosemide against continuous broadband noise has been compared [10].

## Conclusions

In different experiments designed to assess drugs potentially protecting from impulse noise, there may be different proportions of the two possible injurious components: rapid rise time, causing direct (frank) mechanical lesions which are not amenable to reduction by furosemide, and high noise levels for short durations (furosemide would be able to reduce the active mechanical displacements, leading to lower levels of free radicals ROS). When the rapid rise time is attenuated by the experimental conditions (e.g. reverberations degrading it), the noise exposure is dominated by the high noise level component and furosemide (and other drugs which counteract ROS such as anti-oxidants) can reduce the NIHL. However, when the impulse noise is dominated by a rapid rise time reaching a very high intensity (as in actual M16 firing), and the exposure duration is much longer as in the high kurtosis study (eight hours per day for five days) [22], it is likely that the dominant factor producing the threshold shift is the rapid rise time with direct (frank) mechanical tissue damage which cannot be reduced by drugs such as furosemide.

## Competing interests

The authors declare that they have no competing interests.

## Authors' contributions

CA conducted the study and contributed to the writing. JMW contributed to data collection. LK contributed to the interpretation of the results. HS conceived of the study, participated in the design and drafted the manuscript. All authors read and approved the final manuscript.
